# The RNA N6-Methyladenosine Methyltransferase METTL3 Promotes the Progression of Kidney Cancer via N6-Methyladenosine-Dependent Translational Enhancement of ABCD1

**DOI:** 10.3389/fcell.2021.737498

**Published:** 2021-09-23

**Authors:** Yue Shi, Yanliang Dou, Jianye Zhang, Jie Qi, Zijuan Xin, Mingxin Zhang, Yu Xiao, Weimin Ci

**Affiliations:** ^1^Key Laboratory of Genomic and Precision Medicine, China National Center for Bioinformation, Beijing Institute of Genomics, Chinese Academy of Sciences (CAS), Beijing, China; ^2^University of Chinese Academy of Sciences, Beijing, China; ^3^Department of Urology, Peking University First Hospital, Beijing, China; ^4^Institute of Urology, Peking University, Beijing, China; ^5^National Urological Cancer Center, Beijing, China; ^6^Department of Urology, The Affiliated Hospital of Qingdao University, Qingdao, China; ^7^Department of Pathology, Peking Union Medical College Hospital, Chinese Academy of Medical Sciences, Beijing, China; ^8^Institute for Stem Cell and Regeneration, Chinese Academy of Sciences (CAS), Beijing, China

**Keywords:** METTL3, kidney cancer, m^6^A, ABCD1, cancer progression

## Abstract

The role of N6-methyladenosine (m^6^A)-modifying proteins in cancer progression depends on the cell type and mRNA affected. However, the biological role and underlying mechanism of m^6^A in kidney cancer is limited. Here, we discovered the variability in m^6^A methyltransferase METTL3 expression was significantly increased in clear cell renal cell carcinoma (ccRCC) the most common subtype of renal cell carcinoma (RCC), and high METTL3 expression predicts poor prognosis in ccRCC patients using a dataset from The Cancer Genome Atlas (TCGA). Importantly, knockdown of METTL3 in ccRCC cell line impaired both cell migration capacity and tumor spheroid formation in soft fibrin gel, a mechanical method for selecting stem-cell-like tumorigenic cells. Consistently, overexpression of METTL3 but not methyltransferase activity mutant METTL3 can promote cell migration, spheroid formation in cell line and tumor growth in xenograft model. Transcriptional profiling of m^6^A in ccRCC tissues identified the aberrant m^6^A transcripts were enriched in cancer-related pathways. Further m^6^A-sequencing of METTL3 knockdown cells and functional studies confirmed that translation of ABCD1, an ATP-binding cassette (ABC) transporter of fatty acids, was inhibited by METTL3 in m^6^A-dependent manner. Moreover, knockdown of ABCD1 in ccRCC cells decreased cancer cell migration and spheroid formation, and upregulation of ABCD1 acts as an adverse prognosis factor of kidney cancer patients. In summary, our study identifies that METTL3 promotes ccRCC progression through m^6^A modification-mediated translation of ABCD1, providing an epitranscriptional insight into the molecular mechanism in kidney cancer.

## Introduction

Globally, in 2018, there were an estimated 403,000 new cases of renal cell carcinoma (RCC) and 175,000 deaths (Bray et al., 2018). Inactivation of the von Hippel-Lindau tumor suppressor (pVHL) is the best-known oncogenic event in clear cell renal cell carcinoma (ccRCC) (Hsieh et al., 2017). VHL is critical for targeting the α-subunit of hypoxia-inducible factor (HIF) for oxygen-dependent proteolysis (Maxwell et al., 1999; Jaakkola et al., 2001), thus providing a direct molecular link between VHL-associated tumorigenesis and oxygen sensing via HIF. However, *Vhl* deletion in mice failed to elicit tumor formation (Rankin et al., 2006; Kapitsinou and Haase, 2008), suggesting that additional mechanisms are essential. Previous studies including ours have proven that ccRCC is also an epigenetic disease driven by DNA hypermethylation and aberrant histone modifications (Dalgliesh et al., 2010; Cancer Genome Atlas Research Network, 2013; Chen et al., 2016). Very recently, a third component has emerged: the so-called epitranscriptome which is defined as the chemical modifications of RNA that regulate and alter the activity of RNA molecules. It remains largely unknown whether RNA modifications are involved in kidney tumorigenesis. One study has showed that the mutation of *Vhl* and *TP53*, two most important genes in development of ccRCC is associated with the change of N6-methyladenosine (m^6^A) regulatory genes (Zhou J. et al., 2019), suggesting that m^6^A modification may play an important role in ccRCC. Exploring the epitranscriptomic mechanisms during kidney tumorigenesis may lead to new promising therapeutic strategies.

One of the best-studied RNA modifications linked to cancer is m^6^A, which influences a broad spectrum of functions in RNA metabolism, including RNA stability, splicing, processing, localization, and translation initiation (Fu et al., 2014; Roundtree et al., 2017; Huang et al., 2020a). Actually, m^6^A installed by the m^6^A methyltransferases complex is a dynamical and reversible biological process. m^6^A modifications of a transcript are deposited by “writers,” the methyltransferase complex, which is composed of two core components METTL3 and METTL14 (Bokar et al., 1997; Liu et al., 2014), and other accessory regulatory subunits, such as WTAP, and KIAA1429 (Ping et al., 2014; Schwartz et al., 2014), and catalyzed by the demethylases, or erasers for depositing and removing them, including FTO and ALKBH5 (Jia et al., 2011; Zheng et al., 2013). And it can be recognized by m^6^A-binding proteins known as “readers” such as YTHDF1/2, YTHDC1/2 (Du et al., 2016; Xiao et al., 2016; Hsu et al., 2017).

In addition, METTL3 as the first discovered core methyltransferase subunit, plays a major catalytic role in m^6^A methylation process. It was first discovered in 1997 isolated from a HeLa cell nuclear extract that exhibited methyltransferase activity (Bokar et al., 1997; Liu et al., 2014). As the core catalytic component in the methyltransferase complex, loss of METTL3 disrupts numerous physiological processes such as spermatogenesis (Xu et al., 2017), hematopoiesis (Lee et al., 2019), embryonic development (Aguilo et al., 2015), T cell homeostasis (Li H. B. et al., 2017), and memory formation (Zhang et al., 2018). In addition, METTL3 functions as an oncogene or a suppressor gene in many types of cancers by affecting different m^6^A levels of target RNAs (Cui et al., 2017; Vu et al., 2017; Chen et al., 2018; Cheng et al., 2019; Lan et al., 2019; Wanna-Udom et al., 2020). Although, Li et al. (2017) has reported that low expression of METTL3 was related to activations of adipogenesis and mTOR pathways in ccRCCs, the molecular mechanism of METTL3 in regulating of kidney cancer progression via an m^6^A methyltransferase dependent manner remains largely unclear.

Herein, we compared the m^6^A mRNA profiles between normal and ccRCC cancer tissues and characterized a significant association between m^6^A and kidney cancer progression. Mechanistically, we identified that METTL3 promotes tumor migration and tumor spheroid formation (stem-cell-like tumorigenic cells), and ABCD1 is an m^6^A target of METTL3 to treat or prevent kidney cancer metastases. Collectively, our results indicate that METTL3 is a candidate therapeutic target that compromises stem-like tumorigenic cells.

## Materials and Methods

### Clear Cell Renal Cell Carcinoma Specimens and Cell Lines

Primary ccRCC and adjacent kidney tumor samples from two patients involved in this study were obtained from Peking Union Medical College Hospital after pathologic diagnosis. The study was approved by the Ethics Committee of our institutes. Informed consent was obtained from each participant. The ccRCC cell lines used in this study, A498 and 786-O were cultured in DMEM medium (HyClone), 10% fetal bovine serum (FBS, BI), 100 U/ml penicillin, and 100 μg/ml streptomycin in a humidified atmosphere with 5% CO_2_ at 37°C.

### Establishment of Stable Knockdown and Overexpression Cells

Stable knockdown of target genes was achieved by lentivirus-based short hairpin RNA delivery. The sequences that resulted in successful knock down are shown in Supplementary Table 1. Overexpression cells were established by using a modified pLVX-IRES-ZsGreen plasmid. The wild-type and mutant METTL3 plasmids were kindly provided by Pro. Yungui Yang (Beijing Institute of Genomics, Chinese Academy of Sciences) (Yang et al., 2019). Lentiviruses were packaged in HEK293T cells through cotransfecting every shRNA plasmid with packing vectors (PsPAX2, pMD2.G) by X-tremeGENE^TM^ Transfection Reagents (Roche). The lentivirus particles were harvested at 24 and 48 h and directly infected A498 cells under polybrene for 24 h after passage through 0.45 μm syringe filters (Corning). Then, transfected A498 cells were selected for 7 days using 2 μg/ml puromycin or 10 μg/ml blasticidin.

### RNA Extraction and Quantitative PCR Analysis

Total RNA was extracted by using TRIzol reagent (Invitrogen) following the manufacturer’s protocol. For mRNA expression quantification, 1 μg of total RNA was converted to cDNA using the RevertAid First Strand cDNA Synthesis Kit (Invitrogen). Quantitative real-time PCR using the KAPA SYBR FAST Universal qPCR Kit (Applied Biosystems) was performed on a 7500 Fast Real-time PCR system (Applied Biosystems). Quantitative PCR primers sequences are listed in Supplementary Table 1.

### Western Blot Analysis

Western blot analysis was performed following the standard protocol. Total protein from the cell lines was prepared with ice-cold cell lysis buffer (Beyotime Institute of Biotechnology) and quantified using BCA protein assay reagent (Thermo). The primary antibodies used in this study are shown in Supplementary Table 2.

### Liquid Chromatography-Tandem Mass Spectrometry

In brief, 100–200 ng of mRNA was digested by 0.1 U Nuclease P1 (Sigma) and 1.0 U Calf Intestinal Phosphatase (New England Biolabs) and incubated at 37°C overnight. The sample was then filtered (MW cutoff: 3 kDa, Pall, Port Washington), and subjected to LC-MS/MS. The nucleosides were separated by reversed-phase ultra-performance liquid chromatography on an Agilent C18 column with online mass spectrometry detection using a G6410B triple quadrupole mass spectrometer (Agilent Technologies) in the positive ion mode. The m^6^A levels were calculated as the ratio of m^6^A to A based on the calibrated concentrations according to the standard curve obtained from pure nucleoside standards running with the same batch of samples.

### N6-Methyladenosine ELISA

To detect overall levels of m^6^A in different samples, the m^6^A ELISA (EpiGentek) was performed according to the manufacturer’s instructions. In brief, about 300 ng of total RNA was used as an input. Then RNA samples were captured and detected by spectrophotometer (Bio-Rad) at 450 nm. The level of m^6^A methylation was calculated according to the manufacturer’s instructions.

### Cell Viability Assay

We used CCK8 assay (Lab Lead) to study the impact of METTL3 knock down in A498 and 786-O cell lines. The cells were seeded in 96-well plates in a density of 1000 cells/well and were cultivated at 37°C in 5% CO_2_. The cell viability was then measured at 24, 48, and 72 h time point.

### Transwell Migration Assay

Transwell migration assays were performed using 24-well Transwell inserts with an 8 μm pore size (Corning). Briefly, in total, 1 × 10^4^ cells in 200 μl of culture medium without FBS were added to the upper chamber, while 500 μl of medium supplemented with 10% FBS was added to the lower chamber and incubated for 12–24 h at 37°C. The cells invading the lower chamber were fixed with 4% paraformaldehyde for 30 min, stained with 0.1% crystal violet solution for 30 min at room temperature, and counted under an upright microscope (five fields per chamber).

### Cell Invasion Assay

For cell invasion assay, BioCoat^TM^ Matrigel invasion chamber was used according to the manufacturer’s instruction (Corning). Briefly, 2 × 10^4^ cells were resuspended in 200 μl of DMEM medium without FBS, and seeded in the upper portion of the invasion chamber. The lower portion of the chamber contained 500 μl of medium supplemented with 10% FBS. After 16–24 h, non-invasive cells were removed from the upper surface of the membrane with a cotton swab. The invasive cells on the lower surface of the membrane were stained with crystal violet, and counted in four separate areas with an upright microscope.

### Three-Dimensional Fibrin Gel Culture of Tumor Cells

A 3D fibrin gel culture was performed according to a previously described method (Liu et al., 2012). Briefly, fibrinogen (Sea Run Holdings) was dissolved in T7 buffer (pH 7.4, 50 mM Tris, 150 mM NaCl) to obtain a concentration of 2 mg/ml. The proper volume of the fibrinogen solution and the cell solution were mixed, resulting in 1 mg/ml (90 Pa) fibrinogen. Fifty microliters of cell/fibrinogen mixtures was seeded into each well of a 96-well plate with 1 μl of thrombin (0.1 U/μl) and then incubated at 37°C for 30 min. Finally, 200 μl of DMEM containing 10% FBS and antibiotics was added to the plate. After culture for 1, 3, or 5 days, the cells were counted by an upright microscope.

### Tumor Xenograft Model

A498 cells (1 × 10^6^ cells) were resuspended in 100 μl of PBS and subcutaneously injected into the axillary fossa of nude mice (BALB/c-nude, 4 weeks old). The tumor volume was calculated with the formula *V* = 0.5 *ab*^2^, where *a* is the longest tumor axis and *b* is the shortest tumor axis. The animal protocol was approved by the animal ethics committee of the Beijing Institute of Genomics, Chinese Academy of Sciences.

### m^6^A-RNA Immunoprecipitation Sequencing and MeRIP-qPCR

The MeRIP experiment was performed according to the reported protocols. Briefly, total RNA was extracted by TRIzol reagent (Invitrogen). mRNA was isolated by a Dynabeads^®^ mRNA Purification Kit (Invitrogen) and fragmented to approximately 100 nt by an RNA fragmentation kit (Ambion). The m^6^A primary antibody (Synaptic Systems) was incubated with Pierce^TM^ Protein A Magnetic Beads (Thermo Fisher Scientific) for 1 h at 4°C. The fragmented RNA (∼100 nt) was incubated with the antibody-bead mixture for 4 h at 4°C and then washed five times with IP buffer. The samples were eluted with m^6^A nucleotide solution and purified with the phenol-chloroform method. For high-throughput sequencing, the purified RNA fragments from MeRIP and the input RNA were used for library construction with the KAPA Stranded RNA-Seq Library Preparation Kit and sequenced with Illumina HiSeq X Ten. For MeRIP-qPCR, the relevant enrichment of m^6^A of ABCD1 in each sample was analyzed by RT-qPCR.

### Dual Luciferase Reporter Assay

Cells were seeded into the individual wells of a six-well plate and co-transfected with vectors according to the X-tremeGENE^TM^ Transfection Reagents (Roche) protocol. After 48 h, the firefly and Renilla luciferase activities were measured by a Dual Luciferase Reporter Assay System (Promega). Each group was analyzed in triplicate.

### N6-Methyladenosine-miCLIP–Seq

Single-nucleotide-resolution mapping of m^6^A of A498 cells was carried out according to previously published studies (Chen et al., 2015; Linder et al., 2015) with some modifications. Briefly, the mRNA of A498 cells were purified by Dynabeads mRNA Purification Kit (Life Technologies) and fragmented to about 100 nt by the fragmentation reagent (Life Technologies). 2 μg of fragmented mRNAs were incubated with 5 μg of anti-m6A antibody (Abcam) in 300 μl immunoprecipitation buffer (50 mM Tris, pH 7.4, 100 mM NaCl, 0.05% NP-40) at 4°C for 2 h. The mixture was then irradiated three times with 0.15 Jcm^–2^ at 254 nm by a CL-1000 Ultraviolet Crosslinker (UVP), and then incubated with Dynabeads Protein A (Life Technologies) at 4°C for 2 h. After washing, end-repair and linker ligation, the enriched RNA were isolated from the beads by proteinase K digestion, and extracted by phenol–chloroform. Purified RNAs were reverse transcribed by Superscript III reverse transcriptase (Life Technologies). The cDNA from last step was with size selection on a 6% TBE-Urea gel (Life Technologies), and circularization and re-linearization by CircLigase II (Epicenter) and *Bam*HI (NEB), respectively. Then the cDNA was amplified by AccuPrime SuperMix 1 enzyme (Life Technologies) for 20 cycles and sequenced by Illumina HiSeq X Ten according to the manufacturer’s instructions.

### Bioinformatic Analysis

In order to measure the variance of gene expression, we calculated the coefficient of expression variation by using the standard deviation divided by the mean (Brown, 1998).

Sequencing reads were aligned to the human genome GRCh37/hg19 by HISAT2, and the m^6^A peaks were detected by MACS peak-calling software (version 2.1.2) with the default options except for “–nomodel, –keepdup all.” A stringent cutoff threshold for a *q*-value of 1 × 10^–5^ was used to obtain high-confidence peaks. m^6^A motifs were identified by using Homer software. Differential gene expression was calculated by R package DEseq2 using HT-seq reads of input samples counted by HT-Seq python package (version 0.9.1), with a fold-change cutoff of 2.0 and a *p*-value cutoff of 5 × 10^–2^. Gene ontology (GO) analysis was performed using Metascape (Zhou et al., 2019), and GO terms with *p* < 0.05 were defined as significant. Network analysis was performed using ConsensusPathDB (CPDB) software (Kamburov et al., 2013; Herwig et al., 2016).

### Statistical Analysis

All the data related to cell and animal experiments were evaluated with GraphPad Prism software. Measured data are represented as the mean ± standard deviation (SD). Unpaired parametric two-tailed Student’s *t*-test was used to calculate statistical significance. Overall survival was analyzed with the Kaplan-Meier method using the log-rank test to determine significance. *p*-values < 0.05, *p*-values < 0.01 and *p*-values < 0.001 were considered statistically significant (^∗^, ^∗∗^, ^∗∗∗^).

## Results

### The Expression Variability of N6-Methyladenosine-Modifying Genes, Especially METTL3, Is Increased in Clear Cell Renal Cell Carcinoma

**FIGURE 1:**
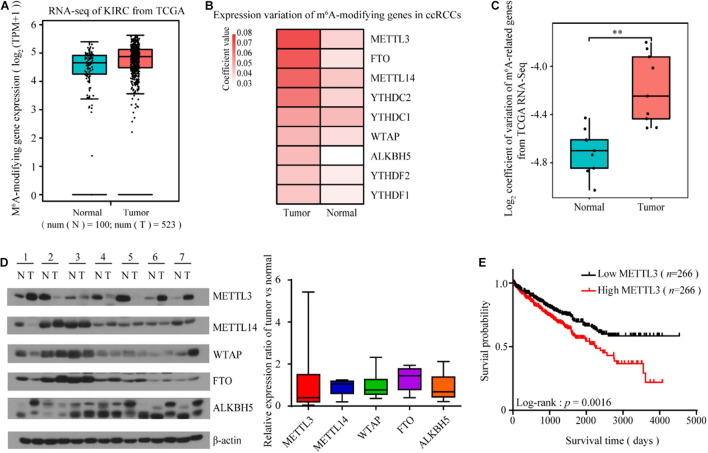
The expression variability of m^6^A-modifying genes is increased in ccRCC, especially METTL3. **(A)** The mean expression of the m**^6^**A-modifying gene set was not significantly changed in the ccRCC tumor tissues compared to the normal tissues from TCGA Data Portal. **(B,C)** The coefficient of expression variation of m^6^A-modifying genes in the ccRCC tumor tissues compared to the normal tissues (unpaired two samples Wilcoxon test). **(D)** The protein levels of m^6^A-modifying proteins in seven paired tumor tissues and matched normal tissues from ccRCC patients was determined by western blots (left). β-actin was used as a loading control. The relative expression ratio of m**^6^**A-modifying proteins between tumors and normal tissues after normalization to β-actin expression (right). **(E)** Kaplan-Meier survival curves using TCGA Data Portal showed that the expression level of METTL3 can predict the clinical outcome of ccRCC patients. *p*-values were calculated by the log-rank test. *n*, the number of cases.

To explore the potential role of m^6^A modification in ccRCC, we first examined the mean expression of m^6^A-modifying genes using RNA-seq data from The Cancer Genome Atlas (TCGA). We found that the mean expression of either the m^6^A-modifying gene set (Figure 1A) or each individual gene (data not shown) did not change significantly in the tumor tissues compared to the normal tissues. However, tumors comprise a heterogeneous collection of cells that can differentially promote progression, metastasis and drug resistance. Intratumor and/or intertumor variability in gene expression has been increasingly shown to be functionally important. Consistent with this scenario, the expression variance of multiple m^6^A-modifying genes especially METTL3, significantly increased during kidney tumorigenesis (Figures 1B,C). Indeed, according to Li et al. (2017) and Zhou et al. (2019) studies, the expression level of METTL3 with high copy number variations showed large heterogeneity in different ccRCC cohorts. To further confirm this finding, we detected the m^6^A-modifying protein levels of seven pairs of tumor and adjacent normal tissues. The expression of m^6^A-related proteins between the normal and tumor tissues varied among the patients, and the variance of METTL3 was the most dramatic (Figure 1D). Kaplan-Meier analysis showed that patients with increased METTL3 expression had a poor overall survival in TCGA Kidney Clear Cell Carcinoma (TCGA-KIRC) cohort (Figure 1E), indicating its role in promoting kidney tumorigenesis.

### METTL3 Promotes Clear Cell Renal Cell Carcinoma Progression in an m^6^A-Methyladenosine Methyltransferase-Dependent Manner

**FIGURE 2:**
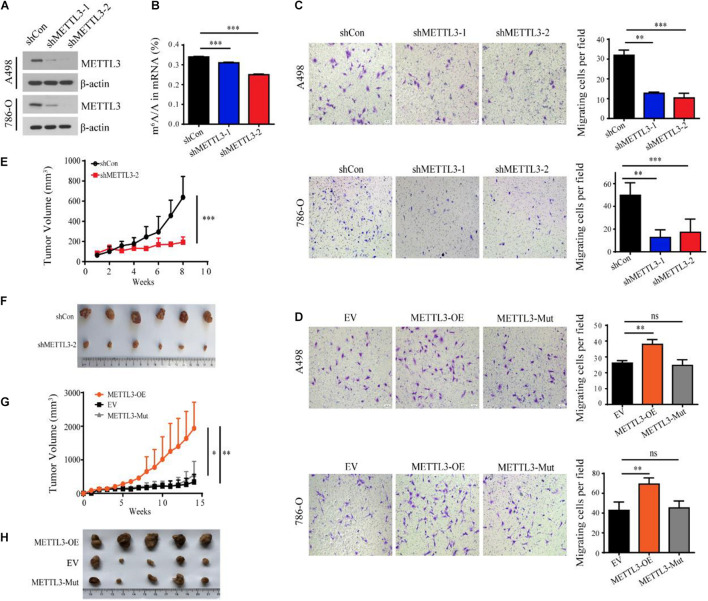
METTL3 promotes ccRCC progression in an m^6^A methyltransferase-dependent manner. **(A)** Western blot of METTL3 in A498 and 786-O cells transduced with lentiviruses expressing scrambled short hairpin RNA (shCon) and two independent shRNAs targeting METTL3 (shMETTL3-1 and shMETTL3-2). β-actin was used as a loading control. **(B)** m^6^A ratio determined by LC-MS/MS in the control and METTL3 knockdown A498 cells. Error bars represent the standard deviation (SD) of three biological replicates. **(C,D)** Transwell assays of A498 and 786-O cells transduced with shRNAs targeting METTL3 **(C)** and METTL3-overexpressing virus **(D)** and quantitatively analyzed (right) (scale bar, 500 μm). EV, control empty virus; METTL3-OE, METTL3-expressing virus; METTL3-Mut, methyltransferase inactivated METTL3-expressing virus. Error bars represent the SD of the mean (*n* = 5). **(E)** Growth curves of xenograft tumors derived from shCon or shMETTL3-2 A498 cells. **(F)** Xenograft tumors formed by shCon or shMETTL3-2 A498 cells in nude mice. **(G)** Growth curves of xenograft tumors derived from A498 cells transduced with METTL3-expressing virus (METTL3-OE), METTL3-Mut-expressing virus (METTL3-Mut) and control empty virus (EV). Error bars represent the SD of the mean (*n* = 5). **(H)** Xenograft tumors formed by METTL3-OE, EV, or METTL3-Mut A498 cells in nude mice.

To further investigate the roles of METTL3 in ccRCC, we established a stable METTL3 knockdown cell line in A498 and 786-O cells (Figure 2A). Liquid chromatography-tandem mass spectrometry (LC-MS/MS) and ELISA assay confirmed that the level of m^6^A in the shMETTL3-1 and shMETTL3-2 cells was significantly decreased (Figure 2B and Supplementary Figure 1A). Functionally, knockdown of METTL3 reduced the cell viability and suppressed their ability in cell migration and invasion (Supplementary Figures 1B,C and Figure 2C). While overexpression of METTL3 but not the kinase inactive mutant METTL3 promoted (Figure 2D). Additionally, stable knockdown of METTL3 effectively suppressed tumor growth as reflected by the significant reduction of tumor size when compared with the shRNA control (Figures 2E,F). But the overexpression of METTL3 rather than the kinase inactive mutant METTL3 could promote the tumor growth *in vivo* xenograft tumor mice models (Figures 2G,H). Therefore, METTL3 is required for kidney cancer progression in an m^6^A methyltransferase-dependent manner.

### METTL3 Is Critical for Stem Cell-Like Tumor-Repopulating Cell Maintenance

**FIGURE 3:**
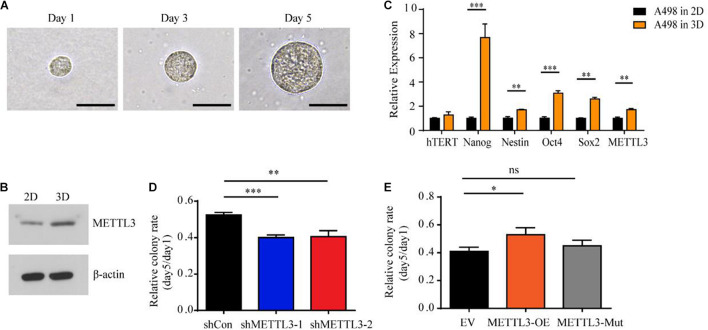
Upregulation of stem cell-associated genes including METTL3 in A498 spheroid cells cultured in 3D fibrin gel. **(A)** A single A498 cell grew into a multicellular tumor spheroid within a 90 Pa 3D fibrin gel during the culture course from Day 1 to Day 5 (scale bar, 500 μm). **(B)** The protein level of METTL3 from A498 cells cultured in 2D rigid dishes or 90 Pa fibrin gels was examined by western blot. β-actin was used as a loading control. **(C)** Stem cell markers and hTERT expression in A498 cells were quantified by RT-qPCR. The same mRNA sample of A498 tumor spheroid cells was used as above. A498 cells cultured in 2D rigid dishes were used as controls. Mean ± SD, *p < 0.05, compared with the 2D cells. **(D,E)** Sphere formation analysis of A498 cells transduced with METTL3-targeting virus **(D)** and METTL3-expressing virus **(E)**, normalized to the number of spheroid cells at day 1. Error bars represent the SD of the mean (*n* = 3).

To further evaluate the functional consequences of the expression variability of METTL3 and identify specific subpopulations that are phenotypically relevant to tumor progression, we used a soft three dimensional (3D) fibrin gel culture system to generate stem cell-like tumor-repopulating cells (TRCs) (Liu et al., 2012; Liu J. et al., 2018). Similarly, A498 ccRCC cells were trapped individually in the gel and grew into spheroid-like shapes resembling stem-like TRCs (Figure 3A). Notably, the protein level of METTL3 was higher in the TRCs than in the 2D rigid dish-cultured cells (Figure 3B). To further test whether the spheroids formed in the soft 3D fibrin gel may share some features of a stem cell, we examined a panel of stem cell markers by RT-qPCR. Consistently, we found that the gene expression of METTL3 was also upregulated in the cells cultured in the soft 3D fibrin gel (Figure 3C). Furthermore, a panel of stem cell makers, Nanog, Nestin, Oct4, and Sox2, were also upregulated in the cells from the soft 3D fibrin gel compared with the control cells (Figure 3C). More importantly, knockdown of METTL3 compromised the colony formation of TRC spheroids, and overexpression of METTL3 but not the kinase inactive mutant METTL3 promoted colony formation (Figures 3D,E). Therefore, METTL3 is also required for TRC colony formation in an m^6^A methyltransferase-dependent manner.

### M^6^A-Methyladenosine Methylomes Are Profoundly Reprogrammed During Clear Cell Renal Cell Carcinoma Tumorigenesis

**FIGURE 4:**
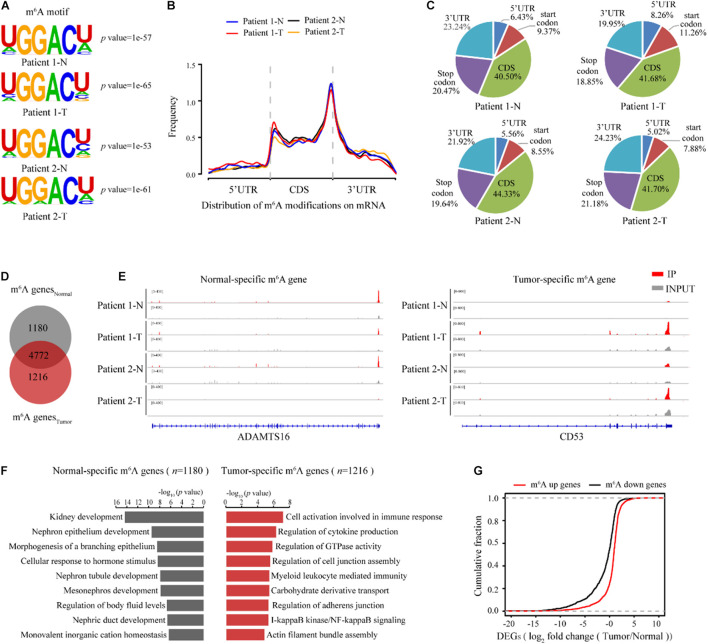
m^6^A methylomes are profoundly reprogrammed during ccRCC tumorigenesis. **(A)** The consensus motif identified from m^6^A-seq peaks in the normal and ccRCC tissues. **(B)** The normalized distribution of m^6^A peaks generated from the normal and tumor tissues across the mRNAs. **(C)** Pie chart of m^6^A peak proportions in the indicated regions in the normal and tumor tissues. **(D)** Venn diagram of the m^6^A-modified genes detected in the normal and tumor tissues. **(E)** Profile of IP and input reads of representative normal-specific and tumor-specific m^6^A peaks in ADAMTS16 and CD53. Levels were normalized by the number of reads in the normal and tumor samples, and the values on the left side of the track represent the range of IP and input level. **(F)** GO pathway analysis of genes with normal-specific and tumor-specific m^6^A modification. **(G)** Cumulative distribution for the gene expression changes between the normal and tumor tissues for upregulated m^6^A methylation genes (red) and downregulated m^6^A methylation genes (black).

We next sought to determine the m^6^A-dependent mechanism by which METTL3 loss impedes ccRCC progression. First, we evaluated the genome-wide m^6^A methylomes during ccRCC tumorigenesis by m^6^A sequencing (MeRIP-seq) with the tumor and matched normal tissues from two ccRCC patients except the seven patients in Figure 1 and Supplementary Table 3. In both the tumor tissues and the normal tissues of the two patients, m6A-seq analysis identified high overlapping 10,434–13,169 m^6^A peaks from 7,038 to 8,214 m^6^A-modified genes (Supplementary Table 4). Consistent with previous studies, the m^6^A peaks were significantly enriched in the RGACH motif (R = G/A; H = A/C/U) (Figure 4A), and were abundant in coding regions (CDSs), 3′UTRs, and near stop codons in all the samples (Figures 4B,C). Then, to determine the biological relevance of m^6^A modification in ccRCC tumorigenesis, we first overlapped the 5,952 m^6^A genes shared in the normal tissues with the 5,988 modified genes shared in the tumor tissues as shown in Figure 4D. And the 1,180 m^6^A genes that only existed in m^6^A genes_*Normal*_ group were regarded as the normal specific genes, while the 1,216 m^6^A genes that only existed in m^6^A genes_*Tum*__*or*_ group were considered as the tumor specific genes (Figure 4D). The representative tracks of normal or tumor m^6^A specific genes were shown in Figure 4E. Interestingly, gene ontology (GO) enrichment analysis revealed that the 1,180 normal-specific m^6^A genes were involved in maintenance of kidney function, such as kidney development, nephron epithelium development, and regulation of body fluid levels (Figure 4F). The GO terms of the 1,216 tumor-specific genes with m^6^A peaks were multiple cancer-related pathways, such as immune-related pathways, regulation of GTPase activity and cell junction assembly (Figure 4F). To test whether the change in m^6^A modification level is correlated with the change in transcript level, we performed RNA-seq of the two patients (Supplementary Figure 2A). There were more downregulated genes in the tumor tissues than in the normal tissues (Supplementary Table 5 and Supplementary Figure 2B). Intriguingly, the downregulated genes also showed significant enrichment in key pathways for maintenance of kidney function, and the upregulated genes showed enrichments in cancer-related pathways (Supplementary Figures 2C,D). We next examined whether the changes in transcript levels were correlated with the changes in m^6^A modification. As shown in Figure 4G, we identified a positive correlation between the change in the m^6^A modification level and that of the mRNA expression level. Thus, m^6^A targets and transcripts showed dynamically controlled abundance during ccRCC tumorigenesis.

### METTL3 Selectively Targets Genes Including Regulators of Fatty Acid Metabolism

**FIGURE 5:**
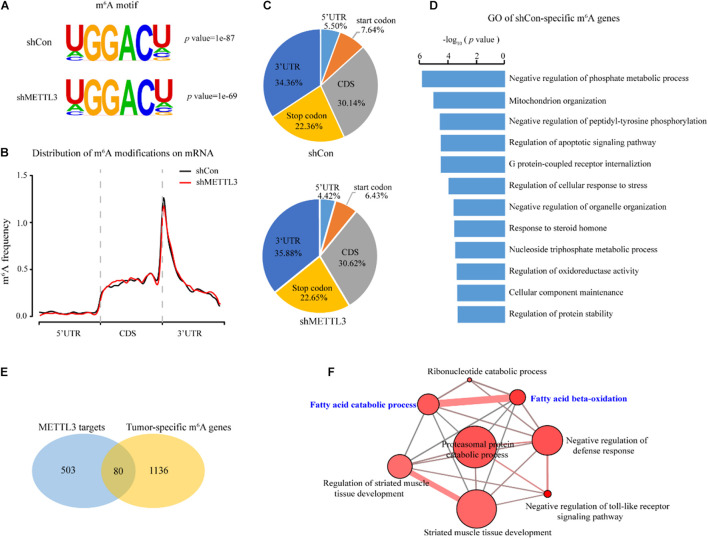
Identification of METTL3 downstream targets in ccRCC. **(A)** Predominant consensus motif identified in the shCon and shMETTL3 A498 cells. **(B)** The normalized distribution of m^6^A peaks across all mRNA transcripts after METTL3 knockdown. **(C)** The fractions of the m^6^A peaks in the control and METTL3 knockdown A498 cells within the indicated regions. **(D)** GO pathway analysis of the candidate METTL3 targets in the ccRCC cell line. **(E)** Schematic of the selection for the downstream targets of METTL3 in the ccRCC cell lines and the ccRCC tissues. **(F)** CPDB analysis of 80 candidate targets. *P*-values were calculated according to Fisher’s exact test, cutoff *p* < 0.01.

To determine which of the deregulated m^6^A modified transcripts could be direct targets of METTL3, we assessed the transcriptome-wide mRNAs with m^6^A in the shCon and shMETTL3 ccRCC cells. Consistent with previous studies, the RGACH motif (R = G/A; H = A/C/U) was highly enriched within m^6^A sites in both the shCon and shMETTL3 cells (Figure 5A). M^6^A peaks in both groups were predominantly located in the CDS, stop codon and 3′UTR (Figures 5B,C). Compared to those in the shCon cells, genes with loss of m^6^A peaks in the shMETTL3 cells were identified as candidate METTL3 targets (shCon-specific genes). In order to obtain the potential targets of METTL3, we overlapped the m^6^A genes in the shCon group with those in the shMETTL3 group, and there were 583 m^6^A genes which were only existed in the shCon group regarded as METTL3 targets (shCon-specific genes). As shown in Figure 5D, these potential METTL3 targets were significantly enriched in cancer-related terms including phosphate metabolism, mitochondrion organization, apoptosis and cellular response to stress. And some of them have been found to be targets of METTL3 in other studies such as SOCS1, GLI1, MAFA, and MGMT (Li et al., 2017; Cai et al., 2019; Wang et al., 2020; Shi et al., 2021). Next, we sought to determine whether the candidate METTL3 targets in the ccRCC cell line are relevant to ccRCC patients. Eighty out of 583 candidate METTL3 targets were preferentially methylated in the tumor tissues of ccRCC patients (Figure 5E). ConsensusPathDB (Kamburov et al., 2013; Herwig et al., 2016) network analysis of these 80 genes further found enrichment for multiple metabolic pathways, such as fatty acid beta-oxidation, fatty acid catabolic process, and ribonucleotide catabolic process (Figure 5F and Supplementary Tables 6, 7).

### ABCD1 Is a Key Downstream Target of METTL3 in Clear Cell Renal Cell Carcinoma

**FIGURE 6:**
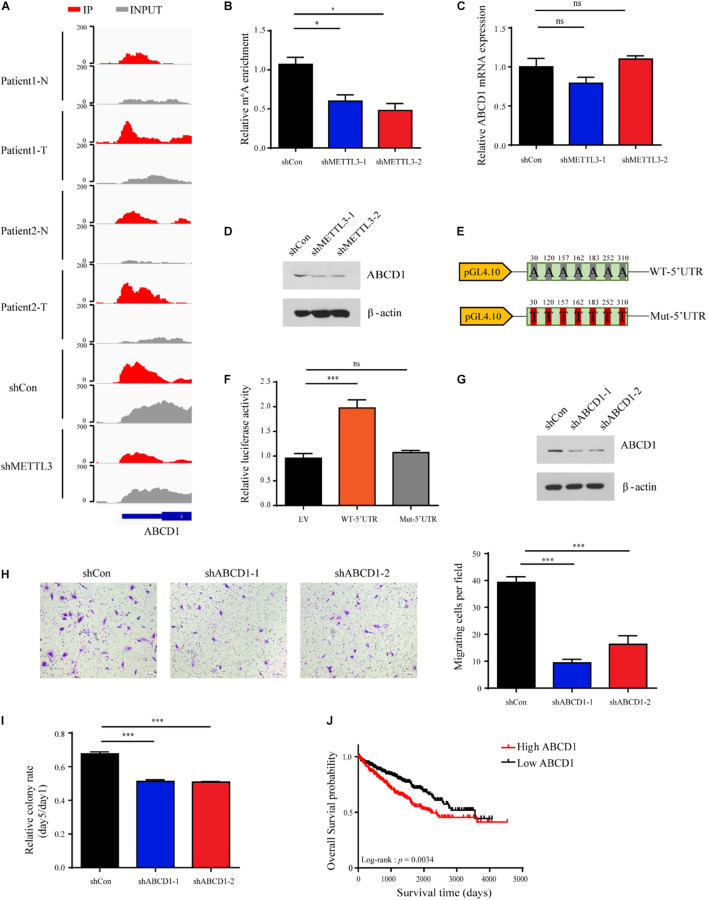
ABCD1 is a key downstream target of METTL3 in ccRCC. **(A)** The read distribution in the 5′UTR of ABCD1 mRNA of the normal and tumor tissues, and the shCon and shMETTL3 A498 cells. The values on the left side of the track represent the range of normalized IP and input levels. **(B)** m^6^A enrichment in the 5′UTR of ABCD1 mRNA in the METTL3 knockdown A498 cells verified by MeRIP-qPCR. Error bars represent the SD of the mean. **(C)** ABCD1 mRNA expression levels in the shCon and shMETTL3 A498 cells verified by RT-qPCR. Error bars represent the SD of the mean. **(D)** ABCD1 protein levels upon knockdown of METTL3 in A498 cells determined by western blotting. β-actin was used as a loading control. **(E)** pGL4.10 luciferase reporter constructs containing fragments of the human ABCD1 5′UTR with wild-type m^6^A sites (WT-5′UTR) or mutant (A-to-T mutation) m^6^A sites (Mut-5′UTR) are shown. The position of the m^6^A sites is numbered relative to the first nucleotide of the 5′UTR. **(F)** Relative luciferase activity in control constructs (EV) or constructs containing the wild-type 5′UTR of ABCD1 (WT-5′UTR) or mutant m^6^A sites of ABCD1 (Mut-5′UTR). Firefly luciferase activity for each construct was first normalized to the co-transfected Renilla luciferase construct and then normalized to the control constructs (EV). **(G)** Western blot of ABCD1 in the A498 cells transduced with lentiviruses expressing scrambled short hairpin RNA (shCon) and two independent shRNAs targeting ABCD1 (shABCD-1 and shABCD1-2). β-actin was used as a loading control. **(H)** (left) and quantitatively analyzed (right) (scale bar, 500 μm). Error bars represent (SD) three biological replicates. **(I)** Sphere-formation analysis of A498 cells transduced with ABCD1-targeting virus, normalized to the number of spheroid cells on day 1. Error bars represent the SD of the mean (*n* = 3). **(J)** Kaplan-Meier survival curves of ccRCC patients based on ABCD1 mRNA expression in TCGA database. Patients were assigned to two subgroups according to the median ABCD1 mRNA expression (log-rank test).

Among the genes enriched in metabolic pathways, we focused on ABCD1, as ABCD1 was identified from multiple metabolic pathways in the ConsensusPathDB network analysis. ABCD1 is a member of the superfamily of ATP-binding cassette (ABC) transporters that is located in the human peroxisome membrane (Tanaka et al., 2002; Morita et al., 2006). As shown in Figure 6A, the m^6^A-seq data demonstrated that the m^6^A peak of ABCD1 in the 5′UTR was markedly increased in tumor tissues and diminished upon METTL3 knockdown in the A498 cells. We also confirmed that the m^6^A abundance decreased after METTL3 knockdown by MeRIP-qPCR in the A498 cells (Figure 6B). However, we found that METTL3 knockdown did not significantly change the mRNA level of ABCD1 (Figure 6C). Since previous studies have found that m^6^A modification in the 5′UTR could promote cap-independent translation (Meyer et al., 2015), we measured the protein level of ABCD1. Western blot assays showed that the protein level of ABCD1 decreased upon METTL3 knockdown (Figure 6D). To further confirm the reduced ABCD1 protein level is due to the methylation of specific sites, we performed single nucleotide resolution m^6^A profiling (miCLIP–seq) of A498 cells and found the 7 m^6^A motifs within the 5′UTR of ABCD1. We then cloned the ABCD1 5′UTR region, including wild-type (WT) or mutant (A-to-T mutation) m^6^A sites into a luciferase reporter vector to identify the function of these m^6^A motifs in the regulation of ABCD1 translation (Figure 6E). As shown in Figure 6F, the luciferase activity was increased in ABCD1 5′UTR wild-type but not in mutant samples. Additionally, knockdown of ABCD1 compromised both tumor migration and tumor sphere formation (Figures 6G–I), which was phenotypically similar to METTL3 knockdown. Moreover, by using TCGA ccRCC RNA-Seq data, we found patients with high ABCD1 expression were associated with poor overall survival (Figure 6J). These results demonstrated that both METTL3 and its downstream targets, such as ABCD1, have potential as therapeutic targets in ccRCC.

## Discussion

Paradoxically, the m^6^A methyltransferase METTL3 was reported to have dual roles in cancer (Huang et al., 2020b), acting as an oncogene in AML (Barbieri et al., 2017), breast (Niu et al., 2019), and liver cancers (Chen et al., 2018), and as a tumor suppressor in glioblastoma (Cui et al., 2017) and endometrial cancers (Liu et al., 2018). However, the direct role of METTL3 via an m^6^A methyltransferase dependent manner in ccRCC remains unclear. Although there has been a report showing that METTL3 impairs ccRCC progression in Caki-1 and Caki-2 cell lines through epithelial to-mesenchymal transition (EMT) and PI3K-Akt-mTOR pathways (Li et al., 2017), other studies identified METTL3 predicts a poor overall survival of ccRCC patients in TCGA datasheet (Chen et al., 2020; Zheng et al., 2020), implying that METTL3 might be an oncogene. In our study, we revealed an important role of METTL3 in regulation of kidney cancer progression. Firstly, we found that the expression variance but not the mean expression level of METTL3 increased significantly during kidney tumorigenesis. Clinical data from TCGA showed that higher expression of METTL3 predicted poor prognosis in ccRCC patients which are consistent with the previously studies. Further functional studies showed that METTL3 was critical for maintaining stem cell-like tumorigenic cells in an m^6^A-dependent manner. MeRIP-seq and GO analysis showed that the m^6^A abundance of METTL3 targets related to metabolic processes is dynamically regulated during kidney tumorigenesis. In particular, ABCD1, an ABC transporter, was methylated in its 5′UTR. Consistent with recent findings (Meyer et al., 2015), we demonstrated that depletion of METTL3 decreased the m^6^A methylation level in the 5′UTR of ABCD1 and reduced the protein level but not the mRNA level. To further demonstrate the regulation of ABCD1 translation efficiency is due to the methylation of specific sites, we performed miCLIP-seq and found 7 m^6^A sites in ABCD1 5′UTR. Mutation of these m^6^A sites in the 5′UTR of ABCD1 has no effect on translation efficiency, but the wild-type one increased the reporter protein level. These results suggested that METTL3 promoted kidney tumorigenesis through enhancing of ABCD1 translation in an m^6^A dependent manner, providing an epitranscriptional insight into the mechanism of kidney cancer progression.

N6-methyladenosine modification could affect the fate of the modified coding and non-coding RNAs in almost all vital bioprocesses, including cancer promotion and progression (Barros-Silva et al., 2020; Huang et al., 2020b; Yang et al., 2020). This study uncovered the crosstalk between complex cancer-associated metabolic reprogramming and epitranscriptomics in kidney cancer. Previous studies including ours have shown that the oncometabolite R-2-hydroxyglutarate (R-2HG) accumulates at high concentrations in ccRCC (Shim et al., 2014; Chen et al., 2016). R-2HG acts as a competitive inhibitor of α-ketoglutarate (α-KG), binding to the active sites of specific enzymes such as α-KG-dependent dioxygenases including DNA “erasers” (TETs) and histone modifiers (Xu et al., 2011) as well as m^6^A “eraser” (FTO and ALKBH5) (Thalhammer et al., 2011; Su et al., 2018). Thus, the m^6^A modification level of the transcripts is susceptible to changes in the concentrations of metabolites and/or oncometabolites. Additionally, the genes with m^6^A modifications are crucial regulators of cell metabolic pathways such as glycolysis, mitochondrial respiratory chain complex activity, and hypoxia. We found that METTL3 selectively targets multiple genes involved in cell metabolism, such as ABCD1. ABCD1 plays a role in the biosynthesis of fatty acids by beta-oxidation and mitochondrial function (van Roermund et al., 2011; Baarine et al., 2015). Morphologically, ccRCC cells show high levels of lipids and glycogens (Gebhard et al., 1987), indicating altered fatty acid and glucose metabolism in the development of ccRCC. Cell-based assays demonstrated that reductive carboxylation of glutamine generates citrate needed for the growth of mitochondrion-defective tumor cells (Metallo et al., 2011; Mullen et al., 2011), and reductive carboxylation of α-KG to citrate for cell growth can be promoted by hypoxia and HIF1 (Wise et al., 2011). Collectively, the crosstalk between cancer-related metabolic reprogramming and m^6^A modifications has a critical role in kidney tumorigenesis.

## Data Availability Statement

The data used in this study can be accessed from the National Genomics Data Center, Beijing Institute of Genomics, Chinese Academy of Sciences, under accession numbers HRA000258 and HRA000259 that are accessible at http://bigd.big.ac.cn/gsa-human.

## Ethics Statement

The studies involving human participants were reviewed and approved by the Ethics Committee of Peking University First Hospital. The patients/participants provided their written informed consent to participate in this study. The animal study was reviewed and approved by Ethics Committee of the Beijing Institute of Genomics, Chinese Academy of Sciences.

## Author Contributions

WC supervised and conceptualized the study. YS and YD performed most of the experiments and bioinformatics analysis. JZ and JQ participated in bioinformatics and statistical analyses. MZ, ZX, and YX were responsible for clinical sample collection and analyses. YS and WC wrote the manuscript. All authors have read and approved the final manuscript.

## Conflict of Interest

The authors declare that the research was conducted in the absence of any commercial or financial relationships that could be construed as a potential conflict of interest.

## Publisher’s Note

All claims expressed in this article are solely those of the authors and do not necessarily represent those of their affiliated organizations, or those of the publisher, the editors and the reviewers. Any product that may be evaluated in this article, or claim that may be made by its manufacturer, is not guaranteed or endorsed by the publisher.

## Supplementary Material

Supplementary Figure 1 METTL3 promotes ccRCC progression related to Figure 2. **(A)** Bar plotting showing the m^6^A level in total RNA in the control and METTL3 knockdown cells by using m^6^A ELISA assay. Error bars represent the SD of three replicates. **(B)** Cell viability of A498 and 786-O cells transduced with shRNAs targeting METTL3. **(C)** Cell invasion assays of A498 and 786-O cells transduced with shRNAs targeting METTL3 (scale bar, 500 μm), and quantitatively analyzed (right), Error bars represent the SD of the mean.

Supplementary Figure 2 Gene expression analysis of normal and ccRCC tissues related to Figure 4. **(A)** Heatmap showing the correlation of RNA-seq data between normal and ccRCC tissues from the 2 patients. **(B)** The volcano plot of the differentially expressed genes (DEGs) between the normal and ccRCC tissues. **(C,D)** GO pathway analysis of the upregulated genes **(C)** and the downregulated genes **(D)** in the ccRCC tissues.

Supplementary Table 1 Sequences of the primers used in this study.

Supplementary Table 2 Antibodies used in this study.

Supplementary Table 3 MeRIP-seq statistics in this work.

Supplementary Table 4 m^6^A-modified gene summary of the two patients in this study.

Supplementary Table 5 Differentially expressed gene summary of the two patients in this work.

Supplementary Table 6 List of the 80 candidates in this work.

Supplementary Table 7 CPDB analysis results of the 80 candidates in this work.
