# Supplementary material for: The RNA N6-Methyladenosine Methyltransferase METTL3 Promotes the Progression of Kidney Cancer via N6-Methyladenosine-Dependent Translational Enhancement of ABCD1
Source: Front Cell Dev Biol. 2021 Sep 23;9:737498. doi: 10.3389/fcell.2021.737498 (PMC8496489; doi:10.3389/fcell.2021.737498)
Supplement: Supplementary Figure 1 — METTL3 promotes ccRCC progression related to Figure 2. (A) Bar plotting showing the m6A level in total RNA in the control and METTL3 knockdown cells by using m6A ELISA assay. Error bars represent the SD of three replicates. (B) Cell viability of A498 and 786-O cells transduced with shRNAs targeting METTL3. (C) Cell invasion assays of A498 and 786-O cells transduced with shRNAs targeting METTL3 (scale bar, 500 μm), and quantitatively analyzed (right), Error bars represent the SD of the mean. [file Data_Sheet_1.docx]

**Supplementary Figures:**


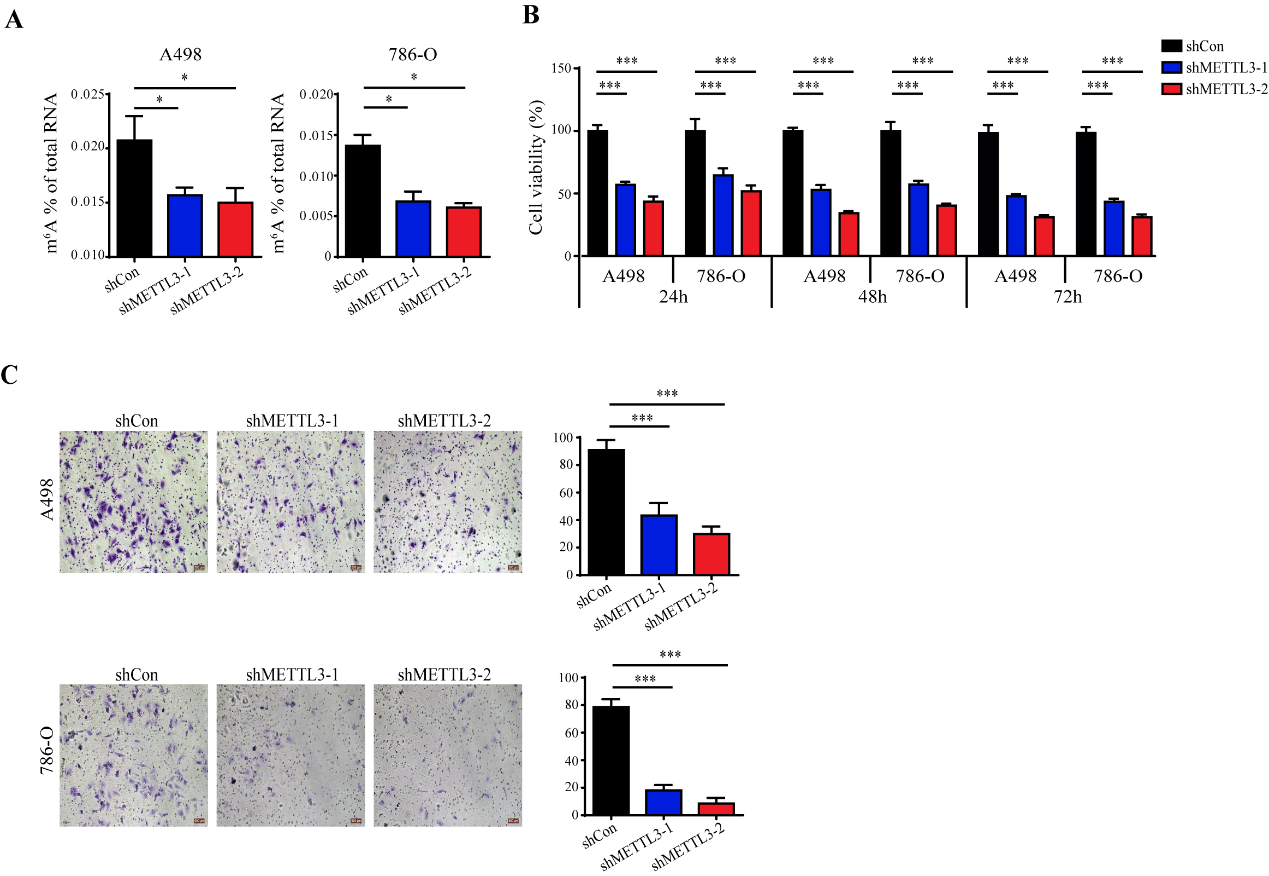


**FIGURE S1. METTL3 promotes ccRCC progression related to Figure 2.** (**A**) Bar plotting showing the m^6^A level in total RNA in the control and METTL3 knockdown cells by using m^6^A ELISA assay. Error bars represent the SD of three replicates. (**B**) Cell viability of A498 and 786-O cells transduced with shRNAs targeting METTL3. (**C**) Cell invasion assays of A498 and 786-O cells transduced with shRNAs targeting METTL3 (scale bar, 500 µm), and quantitatively analyzed (right), Error bars represent the SD of the mean.


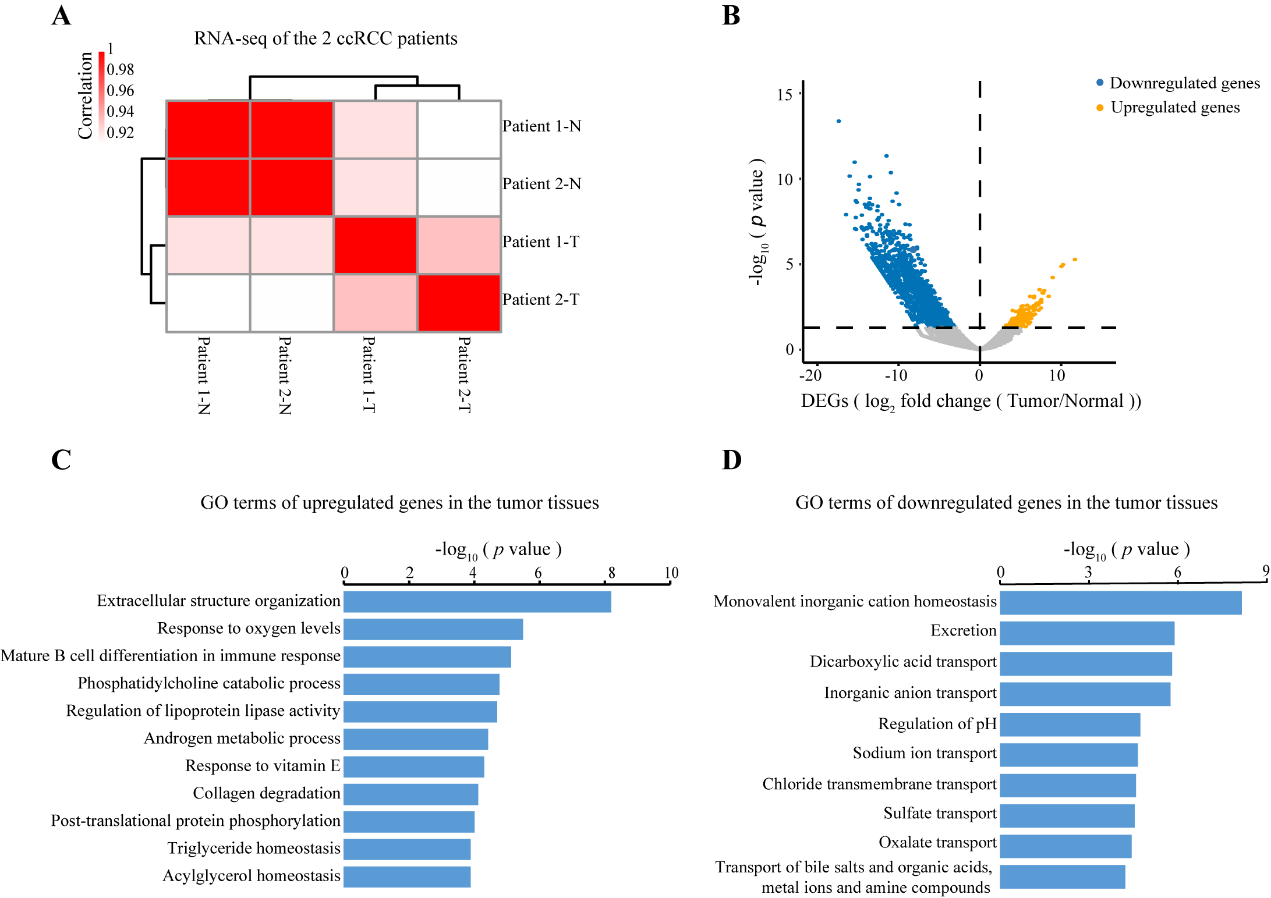


**FIGURE S2. Gene expression analysis of normal and ccRCC tissues related to Figure 4.** (**A**) Heatmap showing the correlation of RNA-seq data between normal and ccRCC tissues from the 2 patients. (**B**) The volcano plot of the differentially expressed genes (DEGs) between the normal and ccRCC tissues. (**C**) and (**D**) GO pathway analysis of the upregulated genes (**C**) and the downregulated genes (**D**) in the ccRCC tissues.
